# Patterns and Safety-Related Aspects of Antidepressant and Anxiolytic Use Among Healthcare Students and University Staff at a Brazilian Public University: A Cross-Sectional Study

**DOI:** 10.3390/pharmacy14050118

**Published:** 2026-08-13

**Authors:** Núbia Vieira Alves, Geórgia Almeida Sant’Ana, Jorge Fernando Carrozza, Gabriela Moraes Oliveira, Carlos Ferreira Santos, Adriana Maria Calvo

**Affiliations:** 1 Department of Biological Sciences, Bauru School of Dentistry, University of São Paulo, Bauru 17012-901, SP, Brazil; nubiavieiraalves@usp.br (N.V.A.); georgia.almeida.santana@gmail.com (G.A.S.); jorgecarrozza@usp.br (J.F.C.); gab.moraes@usp.br (G.M.O.); cfsantos@fob.usp.br (C.F.S.); 2 Hospital for Rehabilitation of Craniofacial Anomalies, University of São Paulo (HRAC/USP), Bauru 17012-901, SP, Brazil

**Keywords:** antidepressants, anxiolytics, psychotropic medications, university students, healthcare personnel, self-reported adverse effects, cross-sectional study

## Abstract

Background/Objectives: The use of antidepressants and anxiolytics has increased worldwide, raising concerns about treatment patterns, medication safety, and rational use. This study aimed to characterize the frequency, utilization patterns, and safety-related aspects of antidepressant and anxiolytic use among healthcare students and university staff at a Brazilian public university. Methods: A cross-sectional study was conducted between September 2024 and June 2025 using an online questionnaire administered to students and staff at the University of São Paulo, Brazil. Descriptive statistics and multivariable logistic regression were applied to characterize medication utilization patterns and evaluate factors associated with antidepressant and/or anxiolytic use. Results: Among the 300 participants, 167 (55.7%) reported antidepressant and/or anxiolytic use during the previous six months. Most users reported treatment for more than one year (59.9%), anxiety as the primary indication (68.3%), and prescription by a healthcare professional (93.4%). A total of 203 medications were reported, with selective serotonin reuptake inhibitors being the most common pharmacological class (42.4%). Among users, 99 (59.3%) reported at least one self-reported adverse effect, most commonly excessive drowsiness, reduced libido/sexual dysfunction, dry mouth, headache, and nausea. No statistically significant independent associations were identified between antidepressant and/or anxiolytic use and the evaluated sociodemographic characteristics. Conclusions: Antidepressant and anxiolytic use was frequently reported and was characterized by predominantly long-term, professionally prescribed treatment and frequent self-reported adverse effects. These findings may inform institutional strategies to improve access to mental healthcare while promoting the rational and safe use of psychotropic medications within university settings.

## 1. Introduction

The use of psychotropic medications, particularly antidepressants and anxiolytics, has increased substantially worldwide over recent decades. Although these medications play a fundamental role in the treatment of anxiety and depressive disorders, their widespread use has also raised concerns regarding prolonged treatment, adverse drug reactions, inappropriate prescribing, and self-medication, making their rational use an important public health priority [1,2,3].

University settings represent a particularly vulnerable environment for mental health problems and psychotropic medication use. The transition to higher education is characterized by substantial academic demands, social adaptation, financial concerns, and uncertainty about future careers. Among healthcare students, these challenges are further intensified by extensive clinical training, early patient contact, and high academic expectations. Together, these factors have been associated with higher levels of anxiety, depression, and psychological distress in national and international studies [3,4,5,6].

However, higher education institutions comprise not only students but also faculty members and administrative staff, all of whom share the same academic environment while facing distinct occupational and psychosocial stressors [3]. Among university employees, heavy workloads, productivity demands, job insecurity, and burnout have consistently been associated with poorer mental health outcomes and greater use of mental healthcare services [7]. Despite growing recognition of mental health challenges among university employees, the available evidence remains focused primarily on undergraduate students, while studies simultaneously evaluating students and university staff within the same academic environment remain scarce [8,9,10,11].

Beyond estimating the prevalence of anxiety and depressive symptoms, medication utilization studies provide complementary information on treatment patterns, prescribing practices, self-medication, treatment adherence, and self-reported adverse effects. By examining how psychotropic medications are used in routine practice, these studies offer a broader understanding of mental healthcare and may contribute to identifying inappropriate medication use and barriers to safe pharmacotherapy [9,12,13].

These issues are particularly relevant in university healthcare settings, where students and healthcare professionals have greater exposure to pharmacological knowledge and easier access to healthcare services, factors that may influence medication-related behaviors. Understanding these patterns is essential for developing institutional strategies that promote rational use of psychotropic medications while ensuring timely access to appropriate mental healthcare [9,12].

Although previous studies have investigated psychotropic medication use among university students, evidence encompassing the broader healthcare academic community, including university staff, remains limited, particularly regarding prescription patterns, treatment duration, self-medication, and medication safety. Addressing this knowledge gap may support the development of institutional mental health policies that promote the rational use of psychotropic medications in higher education settings [9,12]. Therefore, this study aimed to characterize the frequency, utilization patterns, and safety-related aspects of antidepressant and anxiolytic use among healthcare students and university staff at a Brazilian public university, with particular emphasis on treatment duration, prescribing patterns, self-medication, and self-reported adverse effects.

## 2. Materials and Methods

### 2.1. Study Design, Setting, and Participants

This cross-sectional observational study was conducted among members of the healthcare academic community at the Bauru campus of the University of São Paulo (USP), Brazil, between September 2024 and June 2025. The study population comprised undergraduate and graduate students, faculty members, and administrative staff affiliated with the Bauru School of Dentistry (FOB-USP) and the Bauru School of Medicine (FMBRU-USP). For analytical purposes, faculty members and administrative staff were grouped into a single “staff” category because both groups were university employees and the number of participants in each subgroup was insufficient to support separate inferential analyses with adequate precision. This grouping was adopted for analytical feasibility and should not be interpreted as indicating equivalence between the two occupational groups.

Eligible participants were individuals officially affiliated with either institution during the study period who provided electronic informed consent before completing the questionnaire. Because participation was voluntary and recruitment was based on institutional dissemination, a convenience sampling strategy was adopted.

During the study planning phase, the minimum required sample size was estimated based on an institutional population of 1675 individuals, including undergraduate and graduate students, faculty members, and administrative staff from both institutions, according to institutional administrative records. The calculation assumed a 95% confidence level, a 5% margin of error, and an expected prevalence of 50%, resulting in an estimated minimum sample size of 313 participants.

A total of 303 questionnaire responses were received. One response was excluded because electronic informed consent was not provided, and two additional responses were excluded because the participants did not meet the predefined eligibility criteria (no institutional affiliation with FOB-USP or FMBRU-USP). Therefore, the final analytical sample comprised 300 participants.

### 2.2. Data Collection and Questionnaire

Participants were recruited through official communication channels of the USP Bauru campus and institutional social media platforms, including Facebook, Instagram, and WhatsApp. Data collection was conducted between September 2024 and June 2025 using a self-administered electronic questionnaire developed by the research team and administered through Google Forms.

Before data collection, the questionnaire was pilot-tested with 10 individuals from the target population to assess its clarity, comprehensibility, and completion time. Minor wording revisions were implemented before dissemination of the final questionnaire.

Access to the questionnaire was granted only after participants had read and electronically accepted the informed consent form. To prevent multiple submissions by the same participant, Google Forms was configured to restrict responses to one submission per account. In addition, the original response database contained participant identification information, including name, email address, and identification document number. These identifiers were manually cross-checked to identify potential duplicate submissions. No duplicate records were identified. After verification of eligibility and duplicate responses, identifying information was removed, and subsequent data processing and statistical analyses were performed using a de-identified analytical dataset. All questionnaire items were configured as mandatory; therefore, incomplete questionnaires could not be submitted. All questionnaire items, including those related to self-medication practices, were reviewed and approved by the Institutional Research Ethics Committee as part of the approved study protocol. The survey was designed, conducted, and reported in accordance with the Checklist for Reporting Results of Internet E-Surveys (CHERRIES) to enhance the quality, transparency, and reproducibility of web-based survey research [14].

The questionnaire required approximately 10 min to complete and collected data on sociodemographic characteristics, including sex, age, institutional affiliation, academic program, and year of study (when applicable). Participants were asked to report all psychotropic medications used during the previous six months. Information on medication dose or dosage regimen was not collected. Participants were also asked whether their pharmacological treatment had been prescribed by a healthcare professional; this information was recorded at the participant level rather than for each reported medication. When participants reported concomitant use of other psychotropic medications or complementary medicinal products, these were also recorded to provide a more comprehensive characterization of participants’ medication profiles.

The primary outcome was self-reported antidepressant and/or anxiolytic use during the previous six months. Participants were classified as users when they reported the use of at least one medication pharmacologically classified as an antidepressant or anxiolytic based on its established primary pharmacological class. No formal standardized classification system, such as the Anatomical Therapeutic Chemical (ATC) classification, was applied. Concomitantly reported antipsychotics, hypnotics, other psychotropic medications, and complementary or alternative medicinal products were included only for descriptive characterization of medication profiles and did not determine primary-outcome status.

### 2.3. Statistical Analysis

All statistical analyses were performed using Jamovi (version 2.7.37; The Jamovi Project, Sydney, Australia) after verification of data consistency in the final locked analytical dataset. Descriptive statistics were used to summarize the study population, and categorical variables are presented as absolute frequencies and percentages.

Percentages were calculated using the appropriate denominator for each variable (complete-case analysis). Missing or non-classifiable responses were excluded only from the corresponding analyses and are explicitly indicated when applicable.

Associations between antidepressant and/or anxiolytic use and participants’ sociodemographic characteristics were first evaluated using Pearson’s chi-square test or Fisher’s exact test, as appropriate. Variables were selected a priori based on their clinical relevance and because they represent distinct sociodemographic dimensions that may independently influence medication use. Sex, age group, and institutional status (students versus staff) were simultaneously included as independent variables in the multivariable logistic regression model to estimate adjusted odds ratios (ORs) and their corresponding 95% confidence intervals (95% CIs). Self-reported antidepressant and/or anxiolytic use during the previous six months (yes/no) was considered the dependent variable.

Open-ended responses describing self-reported adverse effects were manually reviewed and standardized by grouping synonymous clinical terms into common categories according to predefined coding criteria. The standardization process was performed by a single investigator (N.V.A.) using predefined criteria and subsequently verified against the original dataset to ensure consistency before statistical analysis. Participants could report more than one adverse effect; therefore, event-level frequencies were calculated based on the total number of standardized self-reported adverse-effect events. Less frequently reported events were grouped under Other reactions. No formal assessment of causality, severity, seriousness, adverse-effect mechanism, or potential drug–drug interactions was performed. All statistical tests were two-tailed, and statistical significance was defined as *p* < 0.05.

## 3. Results

The final analytical sample comprised 300 participants and was predominantly composed of women, individuals aged 18–28 years, and students. Detailed sociodemographic characteristics are presented in Table 1.

Among the 300 participants, 167 (55.7%) reported antidepressant and/or anxiolytic use during the previous six months. Most users reported treatment lasting more than one year (59.9%), anxiety as the primary indication (68.3%), and pharmacological treatment prescribed by a healthcare professional (93.4%). The clinical characteristics of antidepressant and/or anxiolytic use are summarized in Table 2.

A total of 203 medications were reported by the 167 users. Of these, 27 participants (16.2%) reported two or more medications, indicating concomitant medication use. Selective serotonin reuptake inhibitors (SSRIs) were the most frequently reported pharmacological class, followed by benzodiazepines and serotonin–norepinephrine reuptake inhibitors (SNRIs) (Table 3). Although the primary outcome focused on antidepressant and/or anxiolytic use, a small number of participants also reported concomitant use of other psychotropic medications or complementary and alternative medicinal products, which were retained for descriptive purposes.

**Figure 1:**
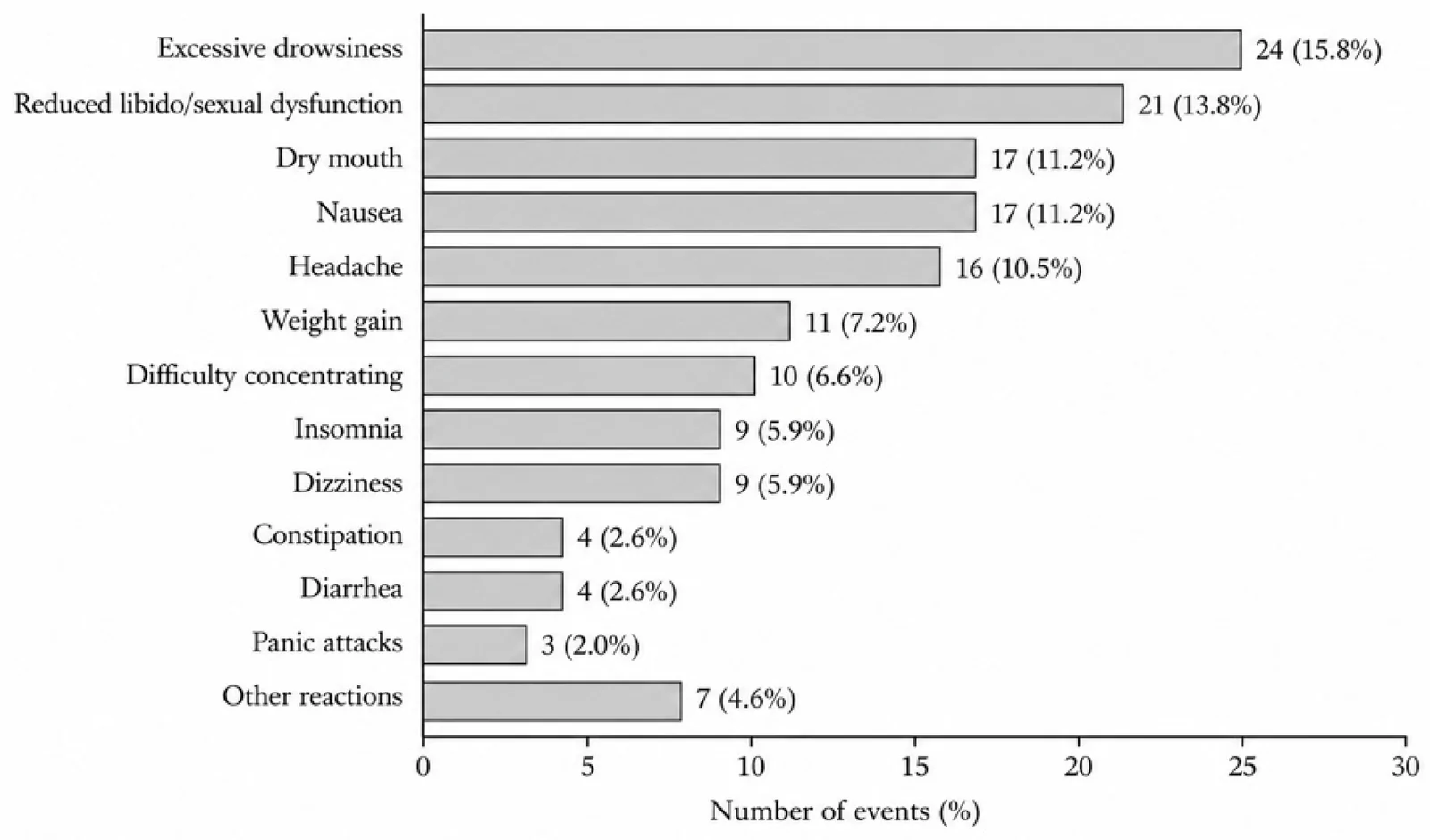
Distribution of standardized self-reported adverse-effect events among participants using antidepressants and/or anxiolytics. A total of 152 standardized self-reported adverse-effect events were identified after data cleaning. Participants could report more than one adverse effect. Less frequently reported events were combined into the category Other reactions.

Among the 167 participants who reported using antidepressants and/or anxiolytics, 99 (59.3%) reported at least one self-reported adverse effect, whereas 65 (38.9%) reported no adverse effects. Three non-informative responses regarding self-reported adverse effects could not be classified and were excluded from the participant-level analysis. After data cleaning and standardization, 152 self-reported adverse-effect events were identified. The most frequently reported adverse-effect events were excessive drowsiness (*n* = 24), reduced libido/sexual dysfunction (*n* = 21), dry mouth (*n* = 17), nausea (*n* = 17), and headache (*n* = 16) (Figure 1).

In the crude analyses, participants aged 29–48 years had higher odds of antidepressant and/or anxiolytic use than those aged 18–28 years (crude OR = 1.84, 95% CI: 1.06–3.20; *p* = 0.032). No statistically significant crude associations were observed for the other category-specific comparisons. Variables considered clinically relevant were subsequently entered into a multivariable logistic regression model (Table 4). Age was categorized into 18–28, 29–48, and ≥49 years to represent young, middle-aged, and older adults while ensuring an adequate number of participants within each category for statistical analysis. Before fitting the multivariable logistic regression model, multicollinearity among the independent variables was assessed using variance inflation factors (VIFs). After adjustment, none of the sociodemographic variables included in the multivariable model showed statistically significant evidence of association with antidepressant and/or anxiolytic use (Table 4).

After adjustment, no statistically significant independent associations were identified between antidepressant and/or anxiolytic use and the evaluated sociodemographic variables (Table 4).

## 4. Discussion

By simultaneously evaluating healthcare students and university staff, the present study provides a comprehensive characterization of antidepressant and anxiolytic use in a Brazilian healthcare academic community. Overall, our findings indicate a high frequency of antidepressant and anxiolytic use, frequent use of selective serotonin reuptake inhibitors (SSRIs), a high frequency of self-reported adverse effects, and no statistically significant independent association between antidepressant and/or anxiolytic use and the evaluated sociodemographic characteristics.

The frequency of antidepressant and/or anxiolytic use observed in the present study (55.7%) was considerably higher than that reported in previous investigations involving university populations. In Brazil, Fares Gianjacomo et al. identified psychotropic medication use in 12.2% of students at a public university, whereas studies conducted in Norway, Italy, and Saudi Arabia reported prevalences ranging from approximately 4% to 30%, depending on the psychotropic drug class evaluated and the study population [9,15,16,17]. These differences should be interpreted cautiously because the present findings were derived from a voluntary convenience sample at a single Brazilian public university and may have been influenced by self-selection bias, self-reported data, and the cross-sectional design. Unlike most previous investigations, the present study simultaneously evaluated healthcare students and university staff and assessed antidepressant and anxiolytic use during the previous six months. One possible explanation is that healthcare academic settings may facilitate recognition of mental health conditions, reduce barriers to seeking professional treatment, and improve access to healthcare services. However, these hypotheses cannot be confirmed by the present cross-sectional study.

Another noteworthy finding was that most participants reported pharmacological treatment lasting longer than one year. Because treatment duration was recorded at the participant level rather than for individual medications, this finding should not be interpreted as indicating prolonged use of any specific pharmacological class, including benzodiazepines. These results suggest that pharmacological treatment in this population was not limited to the management of transient psychological distress. Long-term pharmacological therapy is expected for many chronic anxiety and depressive disorders when clinically indicated and appropriately monitored. The predominance of prolonged treatment observed in the present study is consistent with the worldwide increase in long-term antidepressant use reported over recent decades [18]. Nevertheless, maintenance pharmacotherapy should be accompanied by periodic clinical reassessment to ensure that treatment remains appropriate, effective, and well tolerated over time [19,20].

Anxiety was the primary indication for antidepressant and/or anxiolytic use among the participants, accounting for more than two-thirds of all reported indications. This finding is consistent with the growing recognition of anxiety disorders as one of the leading mental health conditions affecting university populations, particularly among healthcare students, who are exposed to high academic demands, clinical responsibilities, and concerns regarding future professional careers [4,5]. Although depression remains highly prevalent in higher education settings, recent evidence suggests that anxiety symptoms have become increasingly common and frequently represent the main reason for seeking professional mental healthcare and initiating pharmacological treatment [3,8]. Although the present study did not evaluate treatment indications according to institutional status, the predominance of anxiety-related indications in the overall sample suggests that anxiety disorders constitute an important mental health concern throughout the healthcare academic community, extending beyond the student population.

The pharmacological profile observed in the present study was consistent with current recommendations for the treatment of anxiety and depressive disorders. Selective serotonin reuptake inhibitors (SSRIs) accounted for more than 40% of all reported medications, followed by benzodiazepines and serotonin–norepinephrine reuptake inhibitors (SNRIs). The predominance of SSRIs was expected, as these agents remain first-line pharmacological therapy for anxiety disorders and are also recommended as first-line treatment for most depressive disorders because of their favorable balance between efficacy and tolerability [19,21,22]. Benzodiazepines, although effective for short-term symptom control, are generally recommended only for limited periods because of the risks of tolerance, dependence, cognitive impairment, anterograde amnesia, and withdrawal symptoms associated with prolonged use. Historically, concerns regarding these adverse effects have contributed to more restrictive recommendations for benzodiazepine use [23]. The relatively lower frequency of SNRIs and other antidepressants observed in this sample is also consistent with current clinical practice, in which these medications are commonly reserved for patients who do not respond adequately or do not tolerate first-line SSRI therapy [19].

An additional noteworthy finding was the high proportion of participants reporting that antidepressants and/or anxiolytics had been prescribed by a healthcare professional. More than 90% of users reported receiving professional prescriptions, whereas self-medication was uncommon. This finding contrasts with the broader literature on medication use among university students, in which self-medication is frequently reported, particularly for analgesics, anti-inflammatory drugs, and antibiotics [12]. Unlike these therapeutic classes, antidepressants and benzodiazepines are subject to strict regulatory control in Brazil, requiring medical prescription and special dispensing procedures, which may contribute to the low frequency of self-medication observed in the present study [24]. The high proportion of professionally prescribed medications may indicate that many participants had access to formal mental healthcare services. This pattern may also be related to the low frequency of self-medication and the predominance of guideline-recommended pharmacological therapies observed in the present sample.

More than half of the participants using antidepressants and/or anxiolytics reported at least one self-reported adverse effect, reinforcing the importance of continuous monitoring during pharmacological treatment. Excessive drowsiness was the most frequently experienced self-reported adverse effect, followed by reduced libido/sexual dysfunction and dry mouth. These findings are consistent with the well-established safety profiles of SSRIs, SNRIs, and benzodiazepines, which commonly affect the central nervous system, autonomic function, and sexual health [23,25]. Although most of these adverse effects are not life-threatening, they may negatively affect quality of life, daily functioning, and treatment adherence, highlighting the importance of individualized patient counseling and regular clinical follow-up [26]. Because the questionnaire did not link individual symptoms to specific medications and no formal causality assessment was performed, some reported symptoms may also reflect manifestations of the underlying clinical condition rather than medication-related effects. Furthermore, the available data did not permit assessment of potential drug–drug interactions or classification of the reported events according to established adverse drug reaction frameworks. Therefore, these findings should be interpreted as participants’ self-reported experiences during treatment.

Particular attention should be given to dry mouth, which was among the most frequently experienced self-reported adverse effects. Drug-induced xerostomia represents an important clinical concern because persistent salivary reduction is associated with an increased risk of dental caries, periodontal disease, oral candidiasis, halitosis, impaired mastication, and reduced oral health-related quality of life [27,28]. Given that antidepressants and anxiolytics are widely prescribed and are often used for prolonged periods, dentists should routinely investigate psychotropic medication use during anamnesis and implement preventive strategies to minimize oral complications associated with hyposalivation.

Contrary to findings from previous studies, none of the evaluated sociodemographic variables remained independently associated with antidepressant and/or anxiolytic use after multivariable adjustment. Because age group and institutional status represent distinct sociodemographic dimensions, both variables were retained in the multivariable model to evaluate their independent associations with antidepressant and/or anxiolytic use. Although previous studies have frequently reported higher psychotropic medication use among women and younger adults [9,13], these associations were not observed in this sample. These findings should not be interpreted as evidence that antidepressant and/or anxiolytic use is equally distributed across demographic groups. Rather, no statistically significant evidence of association was identified in the present sample.

The absence of significant associations should be interpreted with caution. The relatively homogeneous profile of the study population, composed exclusively of members of a healthcare academic environment, together with the convenience sampling design and the modest sample size, may have reduced the ability to detect small differences between subgroups. These findings highlight the importance of considering mental health needs across the healthcare academic community rather than focusing exclusively on specific demographic groups.

The main strength of this study was the simultaneous characterization of antidepressant and anxiolytic use among healthcare students and university staff within the same academic environment. In addition, all reported medications and self-reported adverse effects underwent a structured data cleaning and standardization process before analysis, enhancing the internal consistency of the dataset. Furthermore, the study evaluated not only the frequency of antidepressant and anxiolytic use but also treatment duration, therapeutic indications, prescription status, pharmacological classes, and self-reported adverse effects, providing a comprehensive overview of medication utilization patterns within a healthcare academic community.

### Study Limitations

Nevertheless, some limitations should be considered. The cross-sectional design precludes causal inferences. Medication use and adverse effects were self-reported and are therefore subject to recall bias. Furthermore, the voluntary convenience sampling strategy may have introduced self-selection bias, as individuals with greater interest in mental health or previous experience with antidepressant and/or anxiolytic use may have been more likely to participate.

In addition, the study sample consisted predominantly of women, younger participants, and students. Although this profile reflects the characteristics of the respondents who voluntarily participated in the survey, it may have influenced the observed frequency of antidepressant and/or anxiolytic use and may limit the generalizability of the findings beyond the study population. Moreover, the relatively homogeneous composition of the sample may have reduced variability across participant subgroups, thereby limiting the ability to detect statistically significant associations between medication use and sociodemographic characteristics.

Because the final sample was slightly smaller than the estimated minimum sample size and recruitment was conducted at a single Brazilian public university, the findings should be interpreted with caution and may not be generalizable to other academic settings. In addition, because adverse effects were not linked to individual medications in the questionnaire, medication-specific safety analyses could not be performed. Future studies should collect medication–event pairings to enable the assessment of adverse-effect profiles according to individual pharmacological classes. Finally, faculty members and administrative staff were analyzed together as a single staff category because of the limited subgroup sizes, which may have obscured potential differences between these occupational groups.

## 5. Conclusions

In conclusion, antidepressant and anxiolytic use was frequently reported by the healthcare students and university staff who participated in this survey at a Brazilian public university, with most users reporting long-term treatment prescribed by a healthcare professional. These findings may inform institutional strategies aimed at promoting the rational use of psychotropic medications while supporting timely access to mental healthcare in university settings.

## Figures and Tables

**Table 1:** Sociodemographic characteristics of the study participants (*n* = 300).

Characteristic	*n* (%)
**Sex**	
Female	222 (74.0)
Male	77 (25.7)
Prefer not to answer	1 (0.3)
**Age group (years)**	
18–28	168 (56.0)
29–48	79 (26.3)
≥49	53 (17.7)
**Academic/occupational status**	
Undergraduate students	126 (42.0)
Dentistry	66 (22.0)
Medicine	45 (15.0)
Speech-Language Pathology/Audiology	15 (5.0)
Graduate students	73 (24.3)
Postdoctoral researchers	5 (1.7)
University employees	96 (32.0)
Administrative staff	53 (17.7)
Healthcare professionals	22 (7.3)
Faculty members	21 (7.0)

**Table 2:** Clinical characteristics of antidepressant and/or anxiolytic use among participants reporting antidepressant and/or anxiolytic use (*n* = 167).

Characteristic	*n* (%)
Treatment duration	
1–30 days	17 (10.2)
1–3 months	17 (10.2)
3–6 months	15 (9.0)
6–12 months	18 (10.8)
>1 year	100 (59.9)
Primary indication *	
Anxiety	114 (68.3)
Depression	36 (21.6)
Other indications	17 (10.2)
Prescription status	
Prescribed by a healthcare professional †	156 (93.4)
Self-medication	11 (6.6)

Values are presented as *n* (%). Percentages were calculated considering participants who reported antidepressant and/or anxiolytic use (*n* = 167). * Primary indications were derived from participants’ open-ended responses and standardized according to predefined classification criteria before analysis. Anxiety-related conditions (including panic disorder, panic attacks, social phobia, obsessive–compulsive disorder, and related disorders) were classified as *Anxiety*. Depression-related conditions were classified as *Depression*. All remaining indications (e.g., attention-deficit/hyperactivity disorder, autism spectrum disorder, insomnia, migraine, grief, menopause-related symptoms, bipolar disorder, and other isolated conditions) were grouped as *Other indications*. † Prescription status was collected at the participant level rather than for each individual medication. Therefore, among participants reporting more than one medication, mixed prescribed and non-prescribed regimens could not be identified.

**Table 3:** Distribution of reported medications according to pharmacological class (*n* = 203 medications).

Pharmacological Class	Reported Medications	*n* (%)
Selective serotonin reuptake inhibitors (SSRIs)	Escitalopram, sertraline, fluoxetine, paroxetine, fluvoxamine	86 (42.4)
Benzodiazepines	Clonazepam, alprazolam, diazepam, bromazepam, lorazepam	36 (17.7)
Serotonin–norepinephrine reuptake inhibitors (SNRIs)	Desvenlafaxine, venlafaxine, duloxetine	32 (15.8)
Other antidepressants	Trazodone, bupropion, nortriptyline, mirtazapine, clomipramine, vortioxetine, agomelatine	27 (13.3)
Other psychotropic medications	Buspirone, quetiapine, pregabalin, zolpidem, risperidone, lisdexamfetamine	14 (6.9)
Complementary and alternative medicinal products	Ansiodoron^®^ (Weleda do Brasil Laboratório e Farmácia Ltda, São Paulo, Brazil), homeopathic preparations, valerian, Bach Rescue floral remedy, cannabidiol	8 (3.9)
Total	—	203 (100.0)

Values are presented as *n* (%). Percentages were calculated considering the total number of reported medications (*n* = 203). Because some participants reported concomitant use of more than one medication, the total number of reported medications exceeds the number of participants. Medications were classified according to their primary pharmacological class. The individual frequencies and pharmacological classification of each reported medication are provided in Supplementary Table S1.

**Table 4:** Participant distribution and crude and adjusted logistic regression analyses of factors associated with antidepressant and/or anxiolytic use among study participants (*n* = 300).

Variable	Users/Total (%)	Crude OR (95% CI)	Crude *p*-Value	Adjusted OR (95% CI)	Adjusted *p*-Value
**Sex**					
Female	128/222 (57.7)	Ref.	—	Ref.	—
Male	39/77 (50.6)	0.75 (0.45–1.27)	0.287	0.71 (0.42–1.20)	0.204
Prefer not to answer ^†^	0/1 (0.0)	—	—	—	—
**Age group (years)**					
18–28	86/168 (51.2)	Ref.	—	Ref.	—
29–48	52/79 (65.8)	1.84 (1.06–3.20)	0.032	1.32 (0.65–2.71)	0.441
≥49	29/53 (54.7)	1.15 (0.62–2.14)	0.654	0.61 (0.21–1.80)	0.373
**Institutional status**					
Students	107/204 (52.5)	Ref.	—	Ref.	—
Staff	60/96 (62.5)	1.51 (0.92–2.48)	0.103	1.87 (0.76–4.61)	0.174

Values are presented as users/total participants within each category (%). Crude odds ratios (ORs) were estimated using univariable logistic regression. Adjusted ORs were estimated using multivariable logistic regression including sex, age group, and institutional status. The dependent variable was self-reported antidepressant and/or anxiolytic use during the previous six months. The regression model included all 300 participants. One participant selected “Prefer not to answer” for sex and was retained in the analysis; because this category contained only one observation, the corresponding regression coefficient was unstable and is therefore not presented. Multicollinearity was assessed using variance inflation factors (VIFs), and no evidence of problematic multicollinearity was identified (all VIFs < 2). ^†^ Included in the regression model; however, because this category contained only one participant, the corresponding coefficient was unstable and is not presented.

## Data Availability

The original contributions presented in this study are included in the article/Supplementary Materials. Further inquiries can be directed to the corresponding author.

## Supplementary Materials

The following supporting information can be downloaded at: https://www.mdpi.com/article/10.3390/pharmacy14050118/s1, Table S1: Reported medications among participants using antidepressants and/or anxiolytics (*n* = 203 medications). Table S2. Standardized self-reported adverse effects among participants using antidepressants and/or anxiolytics (*n* = 152 self-reported adverse-effect events).

## Abbreviations

The following abbreviations are used in this manuscript:

ADHD	Attention-deficit/hyperactivity disorder
CI	Confidence interval
FOB-USP	Bauru School of Dentistry, University of São Paulo
FMBRU-USP	Bauru School of Medicine, University of São Paulo
OCD	Obsessive–compulsive disorder
OR	Odds ratio
SNRI	Serotonin–norepinephrine reuptake inhibitor
SSRI	Selective serotonin reuptake inhibitor
USP	University of São Paulo

## References

[1] Freeman M. (2022). The World Mental Health Report: Transforming mental health for all. World Psychiatry.

[2] United Nations (2015). Transforming Our World: The 2030 Agenda for Sustainable Development. https://sdgs.un.org/goals.

[3] World Health Organization, International Labour Organization (2022). Mental Health at Work: Policy Brief. https://www.ilo.org/publications/mental-health-work.

[4] Sheldon E., Simmonds-Buckley M., Bone C., Mascarenhas T., Chan N., Wincott M., Gleeson H., Sow K., Hind D., Barkham M. (2021). Prevalence and risk factors for mental health problems in university undergraduate students: A systematic review with meta-analysis. J. Affect. Disord..

[5] Ahmed I., Hazell C.M., Edwards B., Glazebrook C., Davies E.B. (2023). A systematic review and meta-analysis of studies exploring prevalence of non-specific anxiety in undergraduate university students. BMC Psychiatry.

[6] Rotenstein L.S., Ramos M.A., Torre M., Segal J.B., Peluso M.J., Guille C., Sen S., Mata D.A. (2016). Prevalence of Depression, Depressive Symptoms, and Suicidal Ideation Among Medical Students: A Systematic Review and Meta-Analysis. JAMA.

[7] Salvagioni D.A.J., Melanda F.N., Mesas A.E., González A.D., Gabani F.L., De Andrade S.M. (2017). Physical, psychological and occupational consequences of job burnout: A systematic review of prospective studies. PLoS ONE.

[8] Mofatteh M. (2021). Risk factors associated with stress, anxiety, and depression among university undergraduate students. AIMS Public Health.

[9] Fares Gianjacomo T.R., Molino Guidoni C., Rodrigues R., de Andrade S.M., Vertuan Rufino J., Girotto E. (2025). Factors associated with the use of psychotropic drugs by students at a brazilian public university. Rev. Peru. Med. Exp. Salud Publica.

[10] Abraham V., Meyer J.C., Mokwena K.E., Duncan E. (2025). Workplace Mental Health Status Among Academic Staff: Psychological Distress, Burnout, and Organisational Culture at a South African University. Behav. Sci..

[11] Hammoudi Halat D., Soltani A., Dalli R., Alsarraj L., Malki A. (2023). Understanding and Fostering Mental Health and Well-Being among University Faculty: A Narrative Review. J. Clin. Med..

[12] Halat D.H., Soltani A., Dalli R., Alsarraj L., Malki A. (2020). Prevalence of self-medication in university students: Systematic review and meta-analysis. East. Mediterr. Health J..

[13] Morris M.R., Hoeflich C.C., Nutley S., Ellingrod V.L., Riba M.B., Striley C.W. (2021). Use of psychiatric medication by college students: A decade of data. Pharmacotherapy.

[14] Eysenbach G. (2004). Improving the quality of Web surveys: The Checklist for Reporting Results of Internet E-Surveys (CHERRIES). J. Med. Internet Res..

[15] Almarghalani D.A., Al-Otaibi K.M., Labban S.Y., Fathelrahman A.I., Alzahrani N.A., Aljuhaiman R., Jamous Y.F. (2025). Prevalence and Impact of Antidepressant and Anti-Anxiety Use Among Saudi Medical Students: A National Cross-Sectional Study. Healthcare.

[16] Bojanić I., Sund E.R., Bjerkeset O., Sivertsen B., Sletvold H. (2021). Psychological Distress and Use of Psychotropic Drugs Among University Students-the SHoT Study, Norway. Front. Psychiatry.

[17] Fasanella N.A., Custódio C.G., Cabo J.S.D., Andrade G.S., de Almeida F.A., Pavan M.V. (2022). Use of prescribed psychotropic drugs among medical students and associated factors: A cross-sectional study. Sao Paulo Med. J..

[18] Horowitz M.A., Buckman J.E., Saunders R., Aguirre E., Davies J., Moncrieff J. (2025). Antidepressants withdrawal effects and duration of use: A survey of patients enrolled in primary care psychotherapy services. Psychiatry Res..

[19] National Institute for Health and Care Excellence (NICE) (2022). Depression in Adults: Treatment and Management. https://www.ncbi.nlm.nih.gov/books/NBK583074/.

[20] Hu Y., Xue H., Ni X., Guo Z., Fan L., Du W. (2024). Association between duration of antidepressant treatment for major depressive disorder and relapse rate after discontinuation: A meta-analysis. Psychiatry Res..

[21] Katzman M.A., Bleau P., Blier P., Chokka P., Kjernisted K., Van Ameringen M. (2014). Canadian clinical practice guidelines for the management of anxiety, posttraumatic stress and obsessive-compulsive disorders. BMC Psychiatry.

[22] Baldaçara L., Paschoal A.B., Pinto A.F., Loureiro F.F., Antonio L.A.V.G., Veiga D.d.L., Almeida T.M., dos Santos D.C., Malloy-Diniz L.F., de Mello M.F. (2024). Brazilian Psychiatric Association treatment guidelines for generalized anxiety disorder: Perspectives on pharmacological and psychotherapeutic approaches. Rev. Bras. Psiquiatr..

[23] Baldwin D.S., Waldman S., Allgulander C. (2011). Evidence-based pharmacological treatment of generalized anxiety disorder. Int. J. Neuropsychopharmacol..

[24] Brasil. Ministério da Saúde. Secretaria de Vigilância Sanitária. Portaria SVS/MS n° 344, de 12 de maio de 1998. Aprova o Regulamento Técnico Sobre Substâncias e Medicamentos Sujeitos a Controle Especial. Brasília, DF. https://anvisalegis.datalegis.net/action/UrlPublicasAction.php?acao=abrirAtoPublico&num_ato=00000344&sgl_tipo=POR&sgl_orgao=SVS/MS&vlr_ano=1998&seq_ato=000&cod_modulo=134&cod_menu=1696.

[25] Jakobsen J.C., Katakam K.K., Schou A., Hellmuth S.G., Stallknecht S.E., Leth-Møller K., Iversen M., Banke M.B., Petersen I.J., Klingenberg S.L. (2017). Selective serotonin reuptake inhibitors versus placebo in patients with major depressive disorder. A systematic review with meta-analysis and Trial Sequential Analysis. BMC Psychiatry.

[26] Papakostas G.I. (2008). Tolerability of modern antidepressants. J. Clin. Psychiatry.

[27] Villa A., Wolff A., Aframian D., Vissink A., Ekström J., Proctor G., McGowan R., Narayana N., Aliko A., Sia Y.W. (2015). World Workshop on Oral Medicine VI: A systematic review of medication-induced salivary gland dysfunction: Prevalence, diagnosis, and treatment. Clin. Oral Investig..

[28] Wolff A., Joshi R.K., Ekström J., Aframian D., Pedersen A.M.L., Proctor G., Narayana N., Villa A., Sia Y.W., Aliko A. (2017). A Guide to Medications Inducing Salivary Gland Dysfunction, Xerostomia, and Subjective Sialorrhea: A Systematic Review Sponsored by the World Workshop on Oral Medicine VI. Drugs R&D.

