# Supplementary material for: Patterns and Safety-Related Aspects of Antidepressant and Anxiolytic Use Among Healthcare Students and University Staff at a Brazilian Public University: A Cross-Sectional Study
Source: Pharmacy (Basel). 2026 Aug 13;14(5):118. doi: 10.3390/pharmacy14050118 (PMC13516260; doi:10.3390/pharmacy14050118)
Supplement: Supplementary file 1 [file pharmacy-14-00118-s001.zip › pharmacy-4456721-supplementary.pdf]

Supplementary Table S1. Reported medications among participants using antidepressants and/or anxiolytics (n = 203 medications).

Medication	Pharmacological class	n (%)
Escitalopram	Selective serotonin reuptake inhibitor (SSRI)	35 (17.2)
Sertraline	Selective serotonin reuptake inhibitor (SSRI)	27 (13.3)
Clonazepam	Benzodiazepine	24 (11.8)
Desvenlafaxine	Serotonin–norepinephrine reuptake inhibitor (SNRI)	21 (10.3)
Fluoxetine	Selective serotonin reuptake inhibitor (SSRI)	17 (8.4)
Trazodone[1]	Serotonin antagonist and reuptake inhibitor (SARI)	8 (3.9)
Venlafaxine	Serotonin–norepinephrine reuptake inhibitor (SNRI)	7 (3.4)
Bupropion	Norepinephrine–dopamine reuptake inhibitor (NDRI)	7 (3.4)
Alprazolam	Benzodiazepine	6 (3.0)
Buspirone	Azapirone anxiolytic	6 (3.0)
Paroxetine	Selective serotonin reuptake inhibitor (SSRI)	5 (2.5)
Duloxetine	Serotonin–norepinephrine reuptake inhibitor (SNRI)	4 (2.0)
Nortriptyline	Tricyclic antidepressant (TCA)	4 (2.0)
Diazepam	Benzodiazepine	3 (1.5)
Mirtazapine	Noradrenergic and specific serotonergic antidepressant (NaSSA)	3 (1.5)
Quetiapine	Atypical antipsychotic	3 (1.5)
Ansiodoron®[2]	Anthroposophic medicinal product	3 (1.5)
Bromazepam	Benzodiazepine	2 (1.0)
Clomipramine	Tricyclic antidepressant (TCA)	2 (1.0)
Fluvoxamine	Selective serotonin reuptake inhibitor (SSRI)	2 (1.0)
Homeopathic preparations	Complementary and alternative medicinal product	2 (1.0)
Pregabalin	Gabapentinoid	2 (1.0)
Vortioxetine	Multimodal serotonergic antidepressant	2 (1.0)
Agomelatine	Melatonergic antidepressant	1 (0.5)
Cannabidiol	Complementary and alternative medicinal product	1 (0.5)
Lisdexamfetamine (Venvanse®)	Central nervous system stimulant	1 (0.5)
Lorazepam	Benzodiazepine	1 (0.5)
Risperidone	Atypical antipsychotic	1 (0.5)
Valerian	Complementary and alternative medicinal product	1 (0.5)
Zolpidem	Non-benzodiazepine hypnotic	1 (0.5)
Bach Rescue floral remedy	Complementary and alternative medicinal product	1 (0.5)
Total	—	203 (100.0)

Values are presented as n (%). Percentages were calculated considering the total number of reported medications (n = 203). Because some participants reported concomitant use of more than one antidepressant and/or anxiolytic together with other psychotropic medications or complementary

medicinal products, the total number of medications exceeds the number of participants. The primary study outcome was defined as self-reported antidepressant and/or anxiolytic use. Additional psychotropic medications and complementary medicinal products reported concomitantly are presented to provide a comprehensive description of participants' medication profiles.

[1] The original database contained the entry “Trazona”, which was standardized to trazodone during data cleaning.
[2] Ansiodoron® is an anthroposophic medicinal product indicated for anxiety-related symptoms and was maintained as reported by participants because it does not have a single conventional active pharmaceutical ingredient comparable to the remaining medications.

Supplementary Table S2. Standardized self-reported adverse effects among participants using antidepressants and/or anxiolytics (n = 152 self-reported adverse-effect events).

**Standardized self-reported adverse effect**	**n**	**%**
Excessive drowsiness	24	15.8
Reduced libido/sexual dysfunction	21	13.8
Dry mouth	17	11.2
Nausea	17	11.2
Headache	16	10.5
Weight gain	11	7.2
Difficulty concentrating	10	6.6
Insomnia	9	5.9
Dizziness	9	5.9
Constipation	4	2.6
Diarrhea	4	2.6
Panic attacks	3	2.0
Other reactions[1]	7	4.6
Total	152	100.0

Values are presented as n (%). Percentages were calculated considering the total number of standardized self-reported adverse-effect events (n = 152). Participants could report more than one self-reported adverse effect. Open-text responses describing self-reported adverse effects were manually reviewed, standardized, and grouped into common clinical categories before analysis.

[1] Other reactions included appetite loss (n = 2), irritability (n = 2), nightmares (n = 1), tremor (n = 1), and apathy/lack of motivation (n = 1). One response ("I cannot say for sure, as I have been taking the medication since 2017") did not describe a self-reported adverse effect and was excluded from the analysis.
